# First Trimester Biomarkers in the Prediction of Later Pregnancy Complications

**DOI:** 10.1155/2014/807196

**Published:** 2014-03-30

**Authors:** Stefan C. Kane, Fabricio da Silva Costa, Shaun Brennecke

**Affiliations:** ^1^Department of Perinatal Medicine, The Royal Women's Hospital, Cnr Grattan Street and Flemington Road, Parkville, VIC 3052, Australia; ^2^Department of Obstetrics and Gynaecology, The University of Melbourne, Parkville, VIC 3010, Australia; ^3^Monash Ultrasound for Women, 15 Murray Street, Clayton, VIC 3170, Australia

## Abstract

Adverse obstetric outcomes, such as preeclampsia, preterm birth, gestational diabetes, and fetal growth restriction, are poorly predicted by maternal history and risk factors alone, especially in nulliparae. The ability to predict these outcomes from the first trimester would allow for the early initiation of prophylactic therapies, institution of an appropriate model and location of care, and recruitment of a truly “high risk” population to clinical trials of interventions to prevent or ameliorate these conditions. To this end, development of adequately sensitive and specific predictive tests for these outcomes has become a significant focus of perinatal research. This paper reviews the biomarkers involved in these multiparametric tests and also outlines the performance of these tests and issues regarding their introduction into clinical practice.

## 1. Introduction

It is common practice for pregnancies to be predictively categorised as “low” or “high” risk, based on the perceived likelihood of an adverse neonatal or maternal outcome. The dichotomous simplicity of such a categorisation is immediately appealing but fails to reflect adequately the spectrum of risk that exists for all pregnant patients and does not acknowledge the limited clinical utility of current strategies for the prediction of obstetric risk, especially among nulliparae. Routine antenatal investigations generally aim to identify concurrent conditions of obstetric relevance, such as thalassaemia, anaemia, and vertically transmissible maternal infections, such as syphilis [1]. First trimester screening for fetal aneuploidy represents the most commonly employed test in early gestation for the prediction of a later pregnancy complication, namely, the delivery of an infant with a chromosomal anomaly. The principles that underpin these multiparametric tests for aneuploidy have informed the recent development of screening strategies using multiple biomarkers in early gestation for the prediction of other later pregnancy complications [2], such as preeclampsia, spontaneous preterm birth, gestational diabetes, and fetal growth restriction. In addition to outlining the rationale for predictive testing in the first trimester, this paper aims to review the composition and performance of testing strategies for these four entities, which are cumulatively responsible for a significant proportion of adverse perinatal and maternal outcomes. For the purpose of this review, a biomarker is considered to be any objectively measured and evaluated characteristic that reflects normal or pathogenic biological processes [3].

## 2. The Rationale for Screening 

The capacity in early gestation to predict later pregnancy complications allows for

- the early commencement of proven prophylactic therapies (pharmacological and otherwise) to reduce the risk of the adverse outcome in question;
- institution of an appropriate model of antenatal care and level of clinical surveillance;
- recruitment of a truly “high risk” population to trials of interventions intended to prevent or mitigate specific adverse outcomes.

Pregnancy is a domain in which past outcomes do, to a large extent, predict future risk. Among the strongest risk factors for preeclampsia, preterm birth, gestational diabetes, and fetal growth restriction is a history of these conditions in a prior gestation, as outlined in Table 1.

However, such information is of little benefit to a first-time mother's pregnancy complicated by these adverse outcomes, which may have resulted in significant maternal and perinatal morbidity, if not mortality. Trying to predict such adverse events on the basis of maternal factors alone is of limited utility in primigravidae. For example, for the prediction of preeclampsia in nulliparae, an algorithm incorporating maternal factors such as body mass index, mean arterial pressure, and family history yields only a 37% detection rate for a 10% false positive rate [16]. These limitations have prompted research into more sophisticated predictive tests for adverse pregnancy outcome.

Abnormal levels of maternal serum analytes assessed in conventional aneuploidy screening have long been acknowledged to have a statistically significant association with adverse obstetric outcome in euploid pregnancies. For instance, a pregnancy-associated plasma protein A (PAPP-A) of less than 0.42 multiples of the median (i.e., less than the 5th centile) in the first trimester has an adjusted odds ratio of 2.81 (95% CI 2.35–3.35) for low birth weight (less than the 5th centile) [17]. However, its sensitivity is only 12.23%, with a positive predictive value of 9.5%. Indeed, no later pregnancy complication can be predicted with sufficient specificity and sensitivity by any single biomarker. Multiparametric testing—the assessment of multiple parameters in combination (including baseline maternal characteristics)—is required to achieve adequate predictive performance, as is the case with first trimester combined screening for aneuploidy [18].  Table 2 summarises the best-performing multiparametric tests for the common pregnancy complications reviewed in this paper.

## 3. Preeclampsia

Preeclampsia is the commonest serious medical disorder of pregnancy, with a worldwide incidence of 2–8% [19]. The utility of a wide range of biomarkers in predicting this outcome has been investigated, including

- maternal mean arterial pressure [20, 21];
- uterine artery Doppler indices [22, 23];
- markers of placental function, such as PAPP-A and plasma protein 13 (PP13) [24];
- other proteins of placental origin, including inhibin A [25] and activin A [26];
- angiogenic agents, such as placental growth factor (PlGF) and vascular endothelial growth factor (VEGF) [27], and their inhibitors soluble fms-like tyrosine kinase-1 (sFlt-1) [28] and soluble endoglin (sEng) [29].

Only in combination do any of these biomarkers offer sufficient sensitivity and specificity to be of clinical utility in the prediction of preeclampsia. In 2009, researchers from the Fetal Medicine Foundation (UK) evaluated a multiparametric test incorporating maternal factors, mean arterial pressure, uterine artery Doppler pulsatility index, placental growth factor (PlGF), and PAPP-A in 7797 singleton pregnancies. It detected 93% of early-onset preeclampsia for a false positive rate (FPR) of 5% [8]. This approach has recently been validated in an Australian population (*n* = 3066), in which 91.7% of early-onset preeclampsia was detected for a FPR of 10% [9], and in Spain (*n* = 5759), with an 80.8% detection rate for early preeclampsia at a 10% FPR [10]. Some institutions have now introduced this testing strategy into clinical practice and are conducting it at the same time as conventional first trimester aneuploidy screening. Interestingly, just as cell-free fetal DNA in maternal serum is becoming the new “gold standard” screening test for aneuploidy, so too has it been found to have value in predicting preeclampsia, with a recent small retrospective case-control study (*n* = 72) demonstrating a sensitivity and specificity of 100% for the development of this condition [30].

The clinical utility of these predictive tests for preeclampsia is enhanced by the potential availability of prophylactic therapies to ameliorate the condition. Low-dose aspirin (75–100 mg daily) has been shown to reduce the risk of preeclampsia, especially if started in early pregnancy (<16 weeks) [31]. Its capacity to do so among those predicted to be at high risk of this condition on the basis of predictive tests is the subject of ongoing randomised trials [32]. The only other therapy proven to reduce the risk of preeclampsia is calcium [33], although many other agents are under investigation, including low-molecular-weight heparin [34], high-dose folate [35], vitamin D [36], and statins [37].

## 4. Preterm Birth

Spontaneous preterm labour accounts for around 60–70% of all preterm deliveries and thus makes a significant contribution to perinatal morbidity and mortality [38]. The disparate pathogenic processes that result in preterm labour are incompletely understood [39], and as such, its prediction remains challenging. Meta-analyses of 116 biomarkers studied over the last four decades [40], and 30 novel biomarkers investigated over the last ten years [41] have concluded that none perform sufficiently in predicting preterm birth to be clinically useful. Similarly, attempts to devise multiparametric models, incorporating biomarkers such as PAPP-A, PP13, and uterine artery Doppler indices in the first trimester, perform as well as or only marginally better than risk prediction based on maternal characteristics alone [42, 43]. Incorporating measurement of cervical length at 11–13 weeks may improve predictive performance, with one such model predicting 54.8% of all spontaneous preterm births earlier than 34 weeks (at a FPR of 10%) when evaluated in 9974 pregnancies [11], although others have not replicated these findings [44, 45]. This is in contrast to cervical assessment in the second trimester, which has consistently been shown to aid in the prediction of preterm birth [46, 47].

Similarly, current strategies to prevent early spontaneous delivery are limited in scope and effect [39]. Apart from modification of lifestyle factors, therapies with the most evidence include vaginal progesterone supplementation [48] and cervical cerclage [49]. These interventions have mostly been studied in women with a history of preterm birth and/or with a short cervix in the midtrimester. Until the pathogenic pathways for preterm labour are better understood, improved prediction and thus prevention of this outcome will remain an enigma [50], especially for nulliparae with no* a priori* risk factors, and may prove to be optimally instituted in the second rather than first trimester.

## 5. Gestational Diabetes

The worldwide incidence of gestational diabetes mellitus (GDM) is rising [51], and although controversy continues regarding optimal diagnostic thresholds [52], there is clear evidence that its identification and treatment optimise perinatal outcomes [53]. Numerous risk factors for gestational diabetes are well established, including maternal BMI, advancing age, cultural background, history of polycystic ovarian syndrome, and family history of diabetes [54], although some develop impaired glucose tolerance in the absence of any identifiable risk factor. Accurate early prediction of those destined to develop gestational diabetes would allow for the early initiation of measures that may prevent or ameliorate the effects of this condition, such as exercise programs [55] and dietary modifications [56], although further research is required to confirm the specific benefits of these early interventions.

Elevated fasting blood sugar levels (within the range of normoglycaemia) in the first trimester are independently associated with the later development of gestational diabetes, with fasting glucose cut-off levels of 80–85 mg/dL yielding sensitivities of 75–55% and specificities of 52–75% for GDM prediction in one study (*n* = 4876) [57]. Other biomarkers shown to be predictive for GDM include sex hormone binding globulin (SHBG), highly sensitive C-reactive protein (CRP), and adiponectin. A model incorporating SHBG and CRP assessed prior to 15 weeks (*n* = 269) predicted GDM with sensitivity and specificity of 74.07% and 75.62%, respectively, with an overall accuracy of 75.46% [12]. An alternative model, using adiponectin and SHBG in addition to maternal characteristics, demonstrated similar predictive performance in a case-control study of 380 women: 74.1% detection for a 20% false positive rate, compared with 61.6% detection using maternal characteristics alone [13].

## 6. Fetal Growth Restriction

A growth-restricted fetus is one that has failed to reach its growth potential. Many, but not all, of such fetuses will be small for gestational age: less than the 10th centile on population or customised growth charts, depending on local policy. Growth-restricted infants are overrepresented in perinatal morbidity and mortality statistics [58] and have increased lifetime risks of cardiovascular and metabolic disease [59]. Fetal growth restriction (FGR) can arise on account of maternal, placental, or fetal factors, many of which are unmodifiable (e.g., maternal age), or cannot be modified once pregnancy is established. As such, apart from lifestyle modifications such as smoking cessation, few interventions have been identified that can prevent or mitigate the effects of FGR in gravida predicted to be at high risk of the same. Low-dose aspirin (75–150 mg daily) has demonstrated benefit in preventing growth restriction in the absence of preeclampsia when commenced in early gestation: a meta-analysis comparing aspirin commenced at <16 weeks and >16 weeks found a significant difference in relative risk of FGR between the two groups (RR = 0.46 (95% CI 0.33–0.64) versus 0.98 (95% CI 0.88–1.08), *P* < 0.001) [60]. At present, the primary benefit arising from the early prediction of growth restriction is likely to be the adoption of a model of obstetric care that facilitates appropriate clinical and sonographic surveillance.

Given the putative similarities in the placental origins of some aspects of FGR and preeclampsia, it is not surprising that many biomarkers are common to the prediction of both. Doppler analysis of the uterine artery in the first trimester is significantly different in pregnancies destined to develop FGR, with a recent prospective study identifying a correlation between the lowest uterine artery pulsatility index value and subsequent birthweight in an unselected population [61]. Ultrasound can also be used to estimate placental volume in the first trimester, which is significantly smaller in FGR pregnancies [62].

As always, multiparametric testing strategies achieve the best predictive performance. Almost three-quarters (73%) of fetal growth restriction requiring delivery prior to 37/40 was identified in a study (*n* = 32850) using a first trimester screening algorithm incorporating maternal factors and numerous biomarkers, including fetal nuchal translucency (NT) thickness, serum pregnancy-associated plasma protein-A (PAPP-A), free beta-human chorionic gonadotrophin (beta-hCG), mean arterial pressure (MAP), uterine artery pulsatility index (PI), placental growth factor (PlGF), placental protein 13 (PP13), and A disintegrin and metalloprotease (ADAM12) [14]. A subsequently proposed model using a more pragmatic number of biomarkers (maternal characteristics, uterine artery pulsatility index, MAP, PAPP-A, and PlGF) achieved a detection rate for FGR requiring delivery <37 weeks of 55.5% for a 10.9% FPR in a study group of 62052 pregnant women [15].

## 7. Future Directions

The application of these predictive tests to the general population of pregnant women is an example of screening. It is crucial that the principles of screening [63], established in the 1960s and unchanged since, be applied to this domain. In particular, there must be interventions proven to improve outcomes for women and their babies who are screened to be at “high risk” of specific pregnancy complications. Patient and clinician acceptability would be enhanced through the delivery of these tests in an integrated model with well documented performance characteristics and would ideally involve screening for aneuploidy as well. The cost- effectiveness of these tests must be clearly established, and robust economic modelling will be required to justify the allocation of limited public healthcare resources to them. If the costs of these tests are to be borne privately, they run the risk of promoting further health inequity, particularly among those who live some distance from tertiary-level obstetric care, for whom these tests may be of significant potential benefit. Furthermore, the potential impact of commercial imperatives on the timing and clinical context of the introduction of these tests should be acknowledged, anticipated, and managed appropriately.

As with any screening program, participation should be voluntary, and clinicians must remain comfortable caring for patients who exercise their right “not to know” and decline any or all of these screening tests. Finally, the emotional and psychological impact of a “high risk” result can be substantial [64], whether the predicted outcome eventuates or not, and it is important that appropriate resources are in place to support those screened to be high risk.

Once these concerns are addressed, predictive testing strategies in early pregnancy could finally offer a clinically meaningful and accurate distinction between “low” and “high” risk pregnancies, with improved outcomes for both as a result.

## Figures and Tables

**Table 1:** Recurrence of pregnancy complications.

	Relative risk (RR)/odds ratio (OR)	95% Confidence interval (CI)
Preeclampsia [4]	RR 7.19	5.85–8.83
Preterm birth [5]	RR 13.56	11.5–16.0
Gestational diabetes [6]	RR 21.33	19.90–22.86
Fetal growth restriction [7]	OR 8.1	7.8–8.5

**Table 2:** Summary of first trimester multiparametric tests for later pregnancy complications.

Complication	Author	Biomarkers	Detection rate	False positive rate
Preeclampsia	Poon et al., 2009 [8]	MF, MAP, UtA PI, PlGF, and PAPP-A	93%	5%
	Park et al., 2013 [9]	MF, MAP, UtA PI, and PAPP-A	91.7%	10%
	Scazzocchio et al., 2013 [10]	MF, MAP, UtA PI, PAPP-A, and free *β*hCG	80.8%	10%

Preterm birth	Greco et al., 2012 [11]	MF, CRL, and cervical length	54.8%	10%

Gestational diabetes	Maged et al., 2013 [12]	SHBG and CRP	74%	24%
	Nanda et al., 2011 [13]	MF, adiponectin, and SHBG	74.1%	20%

Fetal growth restriction	Karagiannis et al., 2011 [14]	MF, NT, PAPP-A, free *β*hCG, MAP, UtA PI, PlGF, PP13, and ADAM12	73%	10%
	Poon et al., 2013 [15]	MF, UtA PI, MAP, PAPP-A, and PlGF	55.5%	10.9%

MF: maternal factors; MAP: mean arterial pressure: UtA PI: uterine artery Doppler pulsatility index; PlGF: placental growth factor; PAPP-A: pregnancy-associated plasma protein A; *β*hCG: beta human chorionic gonadotrophin; CRL: fetal crown-rump length; SHBG: sex hormone binding globulin; CRP: C-reactive protein; NT: fetal nuchal translucency; PP13: placental protein 13; ADAM12: A disintegrin and metalloprotease.
